# A novel electron transfer flavoprotein dehydrogenase (*ETFDH*) gene mutation identified in a newborn with glutaric acidemia type II: a case report of a Chinese family

**DOI:** 10.1186/s12881-020-00995-2

**Published:** 2020-05-11

**Authors:** Mingcai Ou, Lin Zhu, Yong Zhang, Yaguo Zhang, Jingyao Zhou, Yu Zhang, Xuelian Chen, Lijuan Yang, Ting Li, Xingyue Su, Qi Hu, Wenjun Wang

**Affiliations:** 1 Department of Neonatal screen, Sichuan Provincial Hospital for Women and Children, Chengdu, 610000 Sichuan Province China; 2 Hangzhou Genuine Clinical Laboratory Co. Ltd, 859 Shixiang West Road, Hangzhou, 310007 Zhejiang Province China; 3 Neonatal unit, Sichuan Provincial Hospital for Women and Children, Chengdu, 610000 Sichuan Province China

**Keywords:** Glutaric acidemia type II, Multiple acyl-CoA dehydrogenase deficiency, *ETFDH*, Neonatal-onset

## Abstract

**Background:**

Glutaric acidemia type II (GA II) or multiple acyl-CoA dehydrogenase deficiency (MADD, OMIM 231680) is an inherited autosomal recessive disease affecting fatty acid, amino acid and choline metabolism, due to mutations in one of three genes namely, electron transfer flavoprotein alpha-subunit, ETFA, electron transfer flavoprotein β-subunit, ETFB and electron transfer flavoprotein dehydrogenase, ETFDH. Currently, few studies have reported genetic profiling of neonatal-onset GA II. This study aimed to identify the genetic mutations in a Chinese family with GA II.

**Case presentation:**

We reported a case of GA II with purulent meningitis and septicemia and identified a novel *ETFDH* gene mutation in a female infant. The patient developed an episode of hypoglycemia and hypotonicity on the postnatal first day. Laboratory investigations revealed elevations of multiple acylcarnitines indicating glutaric acidemia type II in newborn screening analysis. Urinary organic acids were evaluated for the confirmation and revealed a high glutaric acid excretion. Genetic analysis revealed two mutations in the *ETFDH* gene (c.623_626 del / c. 1399G > C), which were considered to be the etiology for the disease. The novel mutation c.623_626 del was identified in the proband infant and her father, her mother was carriers of the mutation c.1399G > C.

**Conclusions:**

A novel variant (c.623_626 del) and a previously reported missense (c.1399G > C) in the *ETFDH* gene have been identified in the family. The two variants of *ETFDH* gene identified probably underlie the pathogenesis of Glutaric acidemia type II in this family, and also enlarge *ETFDH* genotype-phenotype correlations spectrum.

## Background

Glutaric acidemia type II (GA II), also known as multiple acyl-CoA dehydrogenase deficiency, is an autosomal recessive inborn error of amino acid and fatty acid metabolism [1]. GA II is a disorder with a heterogeneous etiology. The majority of patients show electron transfer flavoprotein (*ETFA*, *ETFB*) or ETF dehydrogenase (*ETFDH*) gene mutations [2]. However, some researchers recently identified defects in the FAD synthase gene (*FLAD1*) involved in GA II onset [3, 4]. The ETF/ETFDH deficiencies are responsible for multiple defects of the dehydrogenation system because they block not only fatty acid oxidation, but also the oxidation of branched-chain amino acids and glutaryl-CoA on the catabolic pathway of lysine, hydroxylysine, and tryptophan [5]. The genes for ETFα, ETFβ and ETFDH proteins (ETFA, ETFB, and ETFDH, respectively) were mapped to 15p23–25, 19q13.1 and 4q33, respectively. Since one of the three genes is affected in GA II, it is essential to accumulate information on genetic mutations to determine any genotype/phenotype correlation and to identify defective enzymes for an accurate diagnosis/prenatal diagnosis of GA II.

The heterogeneous clinical features of patients with GA II fall into three subclasses: two neonatal-onset forms (types I/II) and a late-onset form (type III) [6], neonatal form type I with congenital anomalies (most commonly cystic or dysplastic kidneys) [1]; neonatal form type II without congenital anomalies; and late onset form with myopathic phenotype and rarely metabolic acidosis [7]. Neonatal onset form is usually fatal and characterized by severe non-ketotic hypoglycemia, metabolic acidosis, excretion of large amounts of fatty acid, and amino acid-derived metabolites with congenital anomalies [8]. In addition to hypoglycemia and metabolic acidosis, routine laboratory findings may include hyperammonemia and elevated liver transaminases, cardiomyopathy may be present in some cases. Pathologic abnormalities include fatty infiltration of the liver, heart, and kidneys [1]. Treatment consists of a diet low in protein and fat together with carnitine, ubiquinone, and riboflavin supplementation. Most neonatal-onset patients may not survive due to progressive deterioration despite aggressive treatment [1]. Several adult-onset GA II cases have been reported with *ETFDH* mutations, almost 190 different variations of this gene have been identified [2, 9–16], but few *ETFDH* mutations have been described in neonatal-onset GA II patients [17, 18]. In the present study, we found a novel variant (c.623_626 del) of the *ETFDH* gene in the proband by targeted capture and sequencing. The proband was diagnosed with neonatal onset GA II and presented with poor feeding, hypotonia, seizures, severe lactic acidosis, purulent meningitis and septicemia and died on the 24 day of life. We present detailed clinical data and further delineate the phenotype associated with this disease.

## Materials and methods

### Blood tandem mass spectrometric analysis

Filter-paper dried blood-spot sample of the patient was pretreated with the NeoBase Non-derivatized MS/MS Kit (Perkin Elmer Life and Analytical Sciences, Turku, Finland), amino acid levels on the dried blood spot was analyzed by liquid chromatography-tandem mass spectrometry (ACQUITY TQD, Waters, Milford, MA, USA).

### Urea organic acidemia analysis

The urine sample was collected and the urine organic acid was analyzed with GC-MS (7890B/5977A, Agilent Technologies, Santa Clara, CA, USA) [19].

### Genotyping by next-generation sequencing (NGS)

Genomic DNA was extracted from dried blood spots or peripheral whole blood of the patient and her parents, and high-throughput sequencing (Illumina Next seq 500) of inherited metabolic diseases-related genes were performed after library preparation, solution hybridization, and beads capture to identify potential disease-causing gene mutations.

### Validation mutations by sanger sequencing

Sanger sequencing was used to validate the identified variants of the proband and her family members. Genomic DNA was amplified using custom oligonucleotide primers. PCR amplification of variants was conducted using Phanta Max Master Mix (Vazyme, China). After the purification of PCR products, sequencing analysis was performed.

### Bioinformatics analysis

We checked the novel variant in frequently used databases such as the Human Gene Mutation Database (http://www.hgmd.cf.ac.uk/ac/index.php), ClinVar (https://www.ncbi.nlm.nih.gov/clinvar/), ExAC consortium (http://exac.broadinstitute.org/), and 1000 Genome Project database (http://www.1000genomes.org/). The variant was further assessed for possible pathogenicity using several bioinformatic programs including SIFT, PolyPhen-2, and MutationTaster. The effect of ETFDH protein domain was predicted by the UniProt database (https://www.uniprot.org/uniprot/Q16134).

### Case presentation

The female proband was born at full-term to nonconsanguineous parents after an uneventful pregnancy as the first child of the family. The infant was delivered by cesarean section due to fetal distress with a 25 years old mother and her family had no history of metabolic diseases. The birth weight was 3650 g and initial physical examination revealed a healthy infant with no dysmorphic features.

The newborn was transferred to the neonatal intensive care unit due to poor sucking and response. Laboratory analyses found hypoglycemia (0.06 mmol/L, normal 3.9–6.1 mmol/L) and metabolic acidosis based on the arterial blood gas results, including pH 7.342, pCO_2_ 17.7 mmHg, pO_2_ 77 mmHg, SO_2_ 95%, and BE− 16 mmol/L (reference ranges: arterial pH 7.35–7.45, pCO_2_ 35–45 mmHg, pO_2_ 60–90 mmHg, SO_2_ 95–98%, and BE 0 ± 3 mmol/L). The hypoglycemia and acidosis were treated with glucose and sodium bicarbonate, respectively. Blood analyzer showed the number of white blood cells and neutrophils was increased, and cerebrospinal fluid albumin protein was also elevated. Combined with laboratory analyses and clinical symptoms of the infant with fever, poor feeding, hypotonia, seizures, she was diagnosed with purulent meningitis and septicemia and treatment of ceftriaxone. Transcranial doppler sonography revealed that hemorrhage has occurred. On the fourth day, she occurred fever again with poor feeding, hypotonia, laboratory results with elevated C-reactive protein, after changed meropenem antibiotics and treatment her temperature back to normal but also with hypotonia, poor response, a brain MRI confirmed arachnoid cyst in this case. After 2 weeks, the patient significantly decreased responsiveness and poor feeding, after that she was discharged from hospital with the request of her family.

An expanded newborn screening (NBS) sample obtained on the third day after birth was reported to be positive for glutaric acidemia type II with elevations of multiple acylcarnitines. In particular, C4 butyrylcarnitine levels were extremely high, accompanied by elevated C5, C6, C8, C10, C14, C16, C18, and C5-DC glutaryl carnitine levels. Second-tier NBS testing and urinary organic acids were performed to confirm the infant was positive for GA II. The acylcarnitine profile of two tests is shown in Table 1. Glutaric acid excretion was extremely high with increased levels of OX-2-acetoacetic acid, isovalanyl glycine-2, 2-hydroxy isobutyric acid-2, adipic acid.

**Table 1:** Dried blood spot acylcarnitine results in each separate test

Blood acylcarnitine concentrations (μmol/L)	Patient first testing	Patient second testing	Reference range
Free carnitine (C0)	9.77	10.98	9–50
Acetylcarnitine (C2)	2.04	8.48	2–50
Propionylcarnitine (C3)	0.08	0.40	0–4
Butyrlycarnitine (C4)	**2.28**	**3.92**	0–0.45
Isovalerylcarnitine (C5)	**0.71**	**0.79**	0–0.4
Glutarylcarnitine (C5DC)	**0.42**	**0.85**	0–0.2
Hexanoylcarnitine (C6)	**0.20**	**0.78**	0–0.1
Octanoylcarnitine (C8)	**0.42**	**1.36**	0.01–0.15
Decanoylcarnitine (C10)	**0.31**	**0.62**	0–0.25
Dodecanoylcarnitine (C12)	**0.74**	**1.17**	0–0.35
Tetradecanoylcarnitine (C14)	**1.69**	**2.62**	0–0.4
Hexadecanoylcarnitine (C16)	**8.46**	**9.63**	0.5–6.5
Octadecenoylcarnitine (C18)	**2.34**	**2.22**	0.25–2

Black bold in the table indicates high acylcarnitine concentrations level compared with reference

**Fig. 1:**
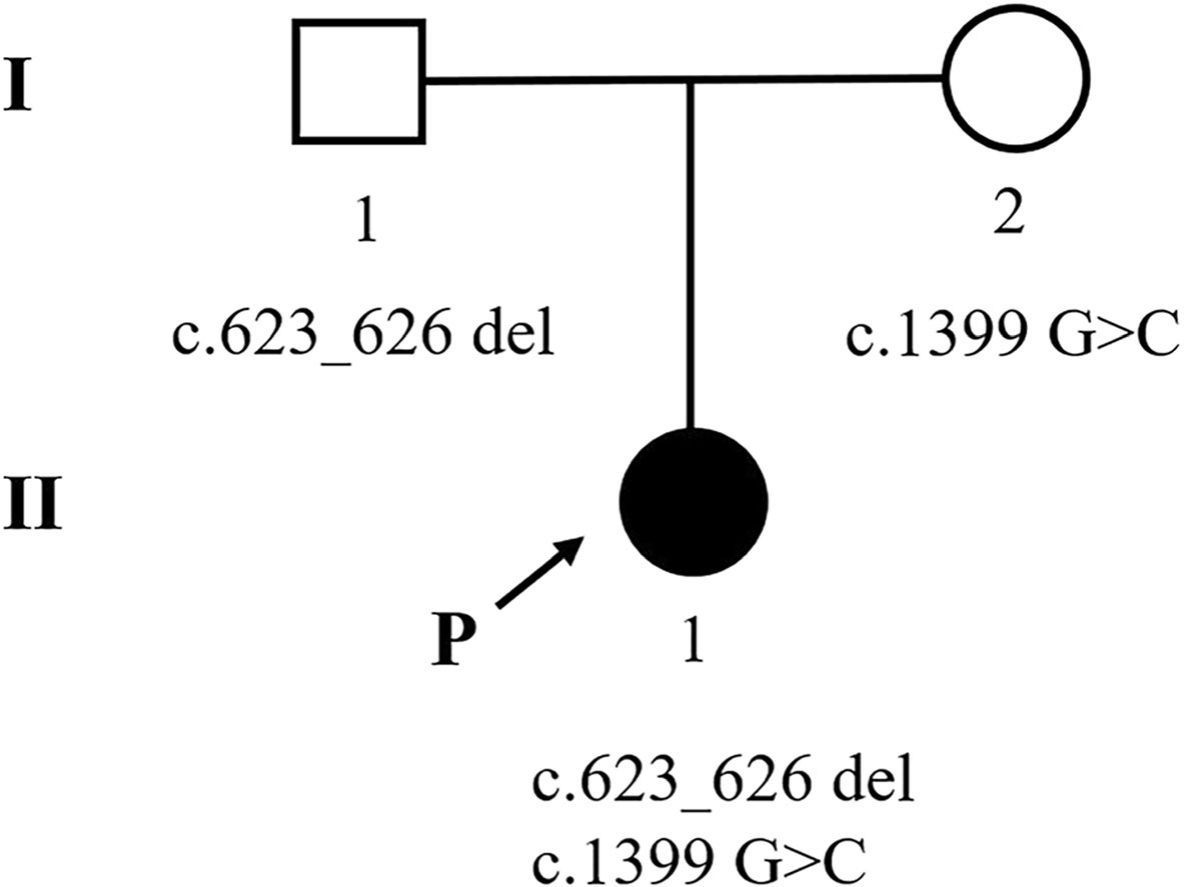
*ETFDH* gene mutations determined by NGS in infant patient and her family. (The filled black symbols represent the affected members and the arrow denotes the proband)

**Fig. 2:**
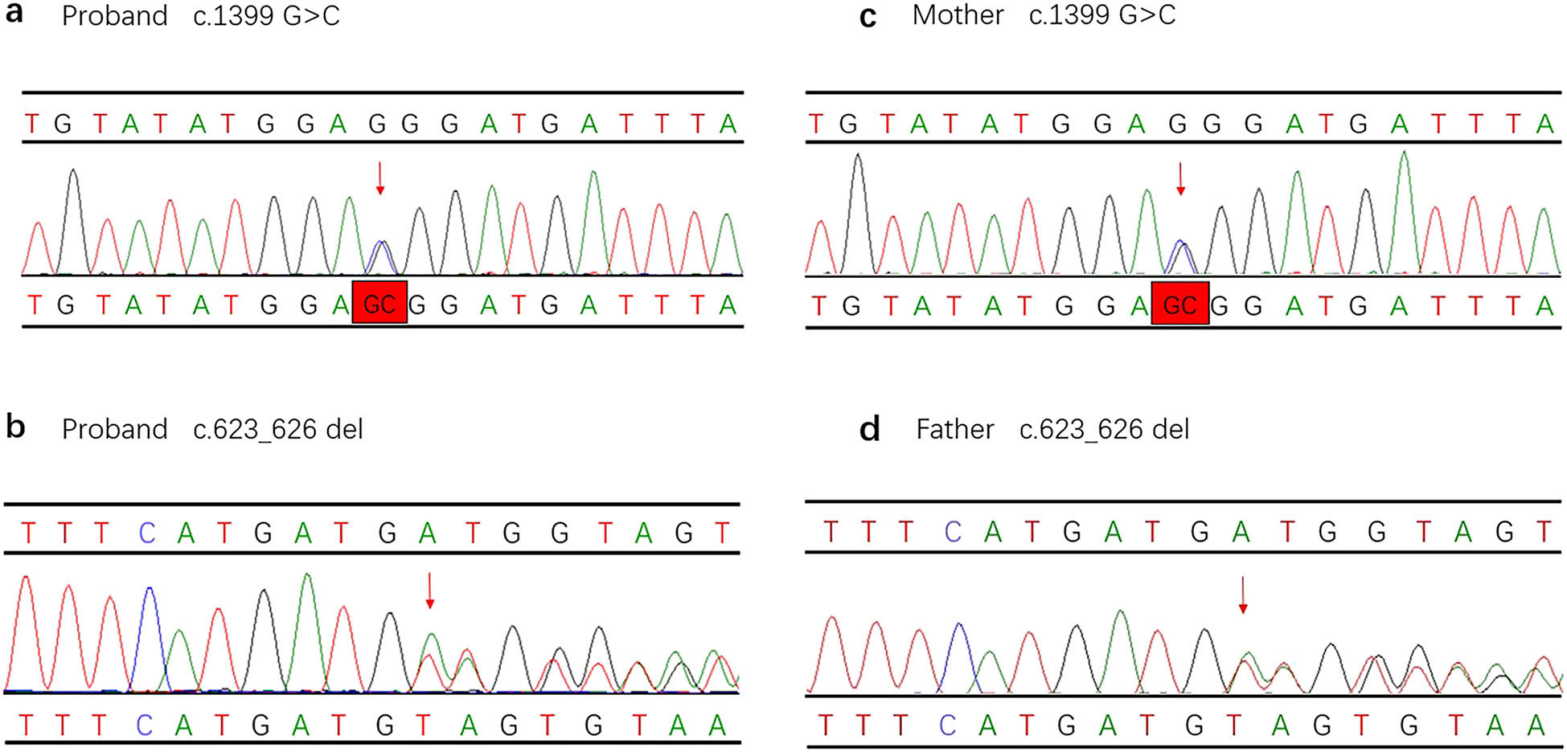
Sequence analysis of *ETFDH* gene mutation in proband and her parents. The heterozygous variant c.1399G > C in exon 11 was identified in proband (**a**) and her mother (**c**); the variant c.623_626 del in exon 6 was identified in proband (**b**) and her father (**d**)

Genetic analysis revealed two different mutations in the *ETFDH* gene of the infant [NM_004453.2 c.1399 G > C (p.G467R) and c.623_626 del (p.D208Vfs*3), compound heterozygote]. The mutation c.1399 G > C has been reported in HGMD (CM093457) and predicted to be deleterious in silico by SIFT, Polyphen-2, and MutationTaster. These two variants have not been previously reported in the databases, ClinVar, ExAC, and 1000 Genomes (Table 2). According to ACMG guidelines, the novel variant c.623_626 del was classified as a likely pathogenic mutation, which was identified in the proband infant and her father, the proband’s mother was carriers of the mutation (c.1399G > C) and these mutations were considered to be the cause of the disease, the pedigree is shown in Fig. 1. These two variants of this family were validated by Sanger sequencing (Fig. 2).

**Table 2:** Analysis and in silico prediction of the *ETFDH* gene variants of the infant

No.	Location	Nucleotide change	Protein change	Parental origin	Type of change	Novelty	SIFT	PolyPhen-2	Mutation Taster	HGMD	ClinVar	Freq in 1000 Genome	Freq in ExAC	Reference
1	Exon 6	c.623_626 del	p.Asp208Val fs*3	paternal	het	novel	N/A	N/A	1	ND	ND	ND	ND	This study
2	Exon 11	c.1399 G > C	p.Gly467Arg	maternal	het	reported	0	1	1	CM 093457	ND	ND	ND	[20]

The reference sequence used in this study was based on the NCBI37/hg19 assembly of the human genome. NM_004453.2 was employed as reference sequence for ETFDH. *ND* no data, *N/A* not available

## Discussion and conclusions

Glutaric acidemia type II, also known as multiple acyl-coenzyme A dehydrogenase deficiency (MADD) was first described in 1976 [19]. GA II is an autosomal recessive disorder resulting from a deficiency of electron transfer flavoprotein (ETF) or ETF dehydrogenase (ETFDH) that manifests from most severe neonatal to late-onset forms. Neonatal-onset form patients may develop severe respiratory failure, cardiomyopathy, hypotonia, metabolic acidosis, and profound hypoglycemia soon after birth, which corresponds with short life expectancy [2]. Variants of the *ETFDH* gene, which contains 13 exons and about 36 kb long, are one cause of GA II. To date, more than 190 *ETFDH* mutations have been reported in Clinvar, with missense mutation as the most common type, of which the major GA II cases were adult-onset, but few disease-causing mutations have been described in neonatal form. Recent reports including this research showed clinical phenotypes in three neonatal-onset patients with *ETFDH* gene mutations (Table 3), F.H. van der Westhuizen et al. [17] reported that a newborn with homozygous variants (c.1067G > A) presented with multiple congenital anomalies including cardiac lesions and hydronephrosis. Vieira et al. [18] described an infant with homozygous variants (c.1141G > C) who presented with profound hypotonia and hepatomegaly, the brain MRI displayed white matter signal abnormalities. Gao et al. [21] reported a Chinese male infant (14 days) with GA II. The results of tandem mass spectrometry showed that C14:1, C8, C6, C10, and C12 increased. Exon sequencing was performed on this infant and revealed compound heterozygous variants in the *ETFDH* gene c.992A > T and c.1450 T > C. After 20 months, the newborn was diagnosed as a late-onset form (type III) without typical clinical symptoms and congenital anomalies. The patient in our study had severe hypoglycemia and with clinical symptoms of fever, poor feeding, hypotonia, the diagnosis was achieved by examination in the CSF and the expanded newborn screening analysis. As a result of these findings, together with MRI examination, the patient was diagnosed with GA II complicated purulent meningitis and septicemia. Purulent or bacterial meningitis is characterized by the invasion and growth of bacteria in the CSF, which is more frequent during the neonatal period than at any other period of life [22]. The clinical features of neonatal bacterial meningitis are nonspecific, CSF study through lumbar puncture is the only way to confirm the diagnosis of meningitis and we examined this case. We genetically analyzed this rare disease in a Chinese female infant and revealed two different mutations (c.1399 G > C / c.623_626 del) in the *ETFDH* gene. The c.1399 G > C mutation was first reported in riboflavin-responsive lipid-storage myopathy male patient, whose age-onset was 12 years [23]. It has been reported in a riboflavin-responsive multiple Acyl-CoA dehydrogenation deficiency late-onset Chinese patient with the c.1399 G > A mutation, western blot analysis revealed a significant reduction of ETF: QO (ETF-ubiquinone oxidoreductase) expression encoded by the *ETFDH* gene, which was more than 90% decrease in mutant protein compared with control [20]. The c.623_626 del mutation was first reported in this study, the variant resulting in the premature termination of the protein decreased. According to the UniProt database analysis, the termination of the protein at position 210 affected functional domains of ETFDH, including FAD/NAD(P)-binding domain (protein position 40–343 and 368–514), 4Fe-4S ferredoxin-type, iron-sulphur binding domain (protein position 577–606), which may affect structure and function of protein and contributed to GA II. Pedigree analysis revealed that her mother and father were carriers of the mutations (c.1399 G > C / c.623_626 del) respectively, the *ETFDH* gene mutation of the infant was compound heterozygote and may affect ETFDH expression resulted in GA II.

**Table 3:** Summary of clinical, biochemical and genetic features associated with *ETFDH* variants of neonatal GA II

	Patient1	Patient2	Patient3
Gender	Male	Male	Female
Origin	Caucasian Afrikaner	Kurdish origin	Chinese
Age of onset/death	Week 1/Day 9	3 months/ 34 months	Day 1/ Day 24
Main clinical features	Intra-uterine growth retardation Multiple congenital anomalies: • Cardiac lesions • Hydronephrosis Riboflavin unresponsive	Profound hypotonia and hepatomegaly. White matter signal abnormalities on brain MRI Riboflavin responsive	Poor sucking and response, fever, hypotonia, seizures Purulent meningitis and septicemia
Routine Biochemistry			
• Metabolic acidosis	Present	Not performed	Present
• Glucose	Normal-↓	↓-Normal	↓
• Ammonia	↑	↑	↑
• Urine ketones	Absent	Not performed	Not performed
• Lactate/pyruvate	Normal-↑	↑	↑
Transaminases	↑GGT, AST	ALT↑	GGT, AST, ALP↑
Creatine kinase	Not performed	normal	Not performed
CBC findings	Pancytopenia	Not performed	White blood cells and neutrophils↑
Metabolic findings			
Acylcarnitines	↑C4-, C5, C5-DC Low free carnitine	↑C8, C10, C12, C14 Normal free carnitine	↑C4-, C5, C5-DC, C6, C8, C10, C12, C14, C16, C18
Organic acids	↑Ethylmalonic acid, Dicarboxylic acids, Glycine conjugates, 2-Hydroxryglutaric acid Lactic acid, Krebs cycle intermediates	↑Glutaric acid, Dicarboxylic aciduria 2-Hydroxyglutaric acid	↑Glutaric acid, OX-2-Acetoacetic acid, 2-Hydroxy isobutyric acid-2, adipic acid, isovalanyl glycine-2,
Amino acids	General amino aciduria with ↑ Sarcosine	↑Threonine, serine, asparagine, glycine, alanine, β-aminoisobutyric acid, and lysine.	↑Citrulline
ETFDH variants	c.1067G > A/ c.1067G > A	c.1141G > C/ c.1141G > C	c.1399 G > C/ c.623_626 del
Reference	F.H. van der Westhuizen et al. [11]	Vieira et al. [12]	Our study

The discovery of novel variants further expands the spectrum of known *ETFDH* mutations in humans and provide molecular evidence for the etiological diagnosis of the patient with GA II as well as for the genetic counseling and prenatal diagnosis in the family.

## Data Availability

All relevant data are included in the manuscript. The datasets used and/or analyzed during the current study are available from the corresponding author upon request.

## Abbreviations

ACMG: American College of Medical Genetics
CSF: Cerebrospinal Fluid
GA II: Glutaric acidemia type II
MRI: Magnetic resonance imaging
NBS: Newborn screening
PDB: Protein data bank
